# Functional Biopolymer Coatings with Nisin/Na-EDTA as an Active Agent: Enhancing Seafood Preservation

**DOI:** 10.3390/foods14122100

**Published:** 2025-06-14

**Authors:** Wladimir Silva-Vera, Sebastián Escobar-Aguirre, Robert Emilio Mora-Luna, Romina L. Abarca

**Affiliations:** 1 Departamento de Biotecnología, Universidad Tecnológica Metropolitana, Las Palmeras 3360, Ñuñoa 7800003, Chile; w.silvav@utem.cl; 2 Departamento de Ciencias Animales, Facultad de Agronomía y Sistemas Naturales, Pontificia Universidad Católica de Chile, Macul, Santiago 7820436, Chile; sebastian.escobar@uc.cl (S.E.-A.); robert.mora@uc.cl (R.E.M.-L.)

**Keywords:** active coating, *Dosidicus gigas* preservation, biopolymer degradation, weight loss control

## Abstract

The increasing demand for reliable food preservation strategies has driven the development of active biopolymer-based films as alternatives to conventional packaging. This study evaluates Nisin/Na-EDTA-enriched alginate and gelatin films for preserving *Dosidicus gigas* (jumbo squid) during refrigerated storage. Films were formulated using alginate, gelatin 220/280 Bloom, and glycerol, and characterized in terms of their mechanical, optical, and biodegradation properties. Their effectiveness for the preservation of squid fillets was tested, focusing on weight loss and color stability during refrigerated storage. The incorporation of Nisin/Na-EDTA significantly modified the film’s properties: elongation at break increased from 4.95% (alginate control) to 65.13% (gelatin 280 active), while tensile strength decreased from 8.86 MPa to 0.798 MPa (alginate). Transparency was reduced by up to 2.5 times in active agent-incorporated alginate films. All films degraded within 14 days under soil exposure, with polysaccharide-based films degrading faster. In refrigerated storage, squid fillets coated with gelatin–alginate films containing Nisin showed reduced weight loss (24.05%) compared with uncoated controls (66.36%), particularly in skin-on samples. Color parameters and whiteness index were better preserved with gelatin-based coatings. These results demonstrate the potential of gelatin–alginate films with Nisin/Na-EDTA as biodegradable, active packaging to extend the shelf life of high-protein seafood.

## 1. Introduction

The consumption of meat and meat products has risen in recent years, with white meat experiencing a particularly higher growth rate compared with red meat, driven by shifting consumer lifestyles [1]. An example of this trend is the increasing demand for seafood, particularly jumbo squid (*Dosidicus gigas*). However, a significant challenge at the industrial level is the short shelf life of seafood, largely due to its high water and protein content. As a high-value product, seafood is particularly susceptible to post-harvest quality deterioration and microbial contamination during storage [2]. Globally, approximately one-third of food is lost or spoiled along the supply chain, preventing its consumption [3]. Shelf life is a critical factor for both industry stakeholders and consumers, particularly in the case of *D. gigas*, which plays a key role in global cephalopod fisheries and represents a significant portion of total squid landings along the coast of Mexico, Perú, and Chile [4,5,6,7].

Jumbo squid is a migratory predatory species distributed throughout the Pacific Ocean, from Alaska to southern Chile [8]. Its distribution is closely associated with high-productivity regions and upwelling systems [9,10,11]. Once captured, most *D. gigas* catches are exported to Asian markets—particularly South Korea and China—where it is valued for its affordability and high protein content, as well as its essential fatty acids, such as eicosapentaenoic acid (EPA), docosapentaenoic acid (DPA), and docosahexaenoic acid (DHA) [12]. Commercially available squid products include fresh-frozen mantle fillets, tentacles, and fins, while byproducts such as viscera and lower-grade squid are processed into animal feed [5]. Given its economic significance, sustainable management practices are crucial to ensuring the long-term viability of this fishery.

The muscle of *D. gigas* is particularly notable for its high protein content, which makes it an appealing ingredient for food innovation [13,14,15]. However, its elevated ammonium levels and distinctive odor present considerable challenges for processing and export [16,17,18]. Non-protein nitrogenous compounds, such as ammonia, negatively impact the sensory characteristics of the product, limiting consumer acceptance in international markets. This highlights the need for more effective preservation techniques. To preserve seafood quality and mitigate spoilage, various preservation methods have been utilized, with flake ice continuing to be the predominant technique in practice [12].

Films and coatings are packaging technologies for food conservation that aim to control and maintain the intrinsic quality of food products, being extensively reported in the literature [19,20,21,22,23]. These materials have a thickness of less than 0.3 mm and are composed of biopolymers and additives dispersed in an aqueous medium [24]. It is relevant to clarify that coatings are applied as thin liquid layers that dry directly on the food surface, while films are pre-formed sheets produced through molding and drying before application [24,25,26]. These technologies have been widely used across different food matrices and are applied using various methods, including brushing, dipping, spraying, and enrobing [27,28,29].

Biopolymers have emerged as a promising alternative to conventional plastic packaging, offering a sustainable solution for food preservation. These materials encompass a diverse range of formulations, including polysaccharides, proteins, lipids, and composite blends, each engineered to meet specific preservation requirements. Among these formulations, the polysaccharide alginate derived from the seaweed species *Phaeophyceae* offers exceptional properties due to its unique gelling, thickening, and film-forming properties. Moreover, it has the ability to interact with calcium ions, forming a semi-permeable membrane. On the other hand, gelatin is obtained through the hydrolysis of collagen, and its film-forming properties—such as high transparency, mechanical strength, and reduced water vapor transmission—make it an excellent alternative as a sustainable solution for food preservation [30]. These functional properties of biopolymers may be enhanced by adding antimicrobial substances in order to extend the shelf life of food products under storage conditions. Among these substances, including essential oils, antioxidants, chemical compounds, and antimicrobial peptides, the food industry has prioritized the use of stable agents considering the operational conditions and GRAS status.

Nisin, a polypeptide bacteriocin produced by *Lactococcus lactis* subsp. *lactis*, is the most extensively characterized bioactive agent against Gram-positive bacteria. Its biological, chemical, biosynthetic, and genetic properties have been well documented which, along with its GRAS (Generally Recognized as Safe) status, makes it a widely used food preservative [31]. While nisin exhibits broad antimicrobial activity, its effectiveness is limited against Gram-negative bacteria, yeasts, and molds [32]. However, studies have shown that the antimicrobial activity of nisin against Gram-negative bacteria can be enhanced through a synergistic effect with chelating agents, such as EDTA. Disodium EDTA (Na-EDTA) is commonly used as a direct food additive, and its enhanced antimicrobial effect against Gram-negative bacteria may be attributed to its ability to bind metal ions in the lipopolysaccharide (LPS) layer of the bacteria [31].

Previous research has shown that an edible coating containing corn zein and *Heracleum persicum* essential oil reduced microbial growth, moisture loss, fat oxidation, and pH variation over time in whey-less cheese over 56 days of cold storage [28]. Likewise, *Malva sylvestris* L. (mallow) flower extract and polysaccharide-based coatings (pectin, xanthan, and carboxymethylcellulose) exhibited strong moisture retention in the loaf crumb while significantly extending the microbiological stability of the product [33].

Further studies have investigated the effects of polymer blending on the functional properties of edible films. Chen et al. (2024) [34] systematically compared the influence of chitosan, carrageenan, and sodium alginate on the optical, mechanical, thermal, and hydrophilic properties of gelatin-based films. The incorporation of chitosan effectively reduced the ultraviolet transmittance, while carrageenan and sodium alginate increased the melting temperature (T_m_) of gelatin film to 92 °C and 84 °C, respectively. However, both additives reduced the hydrophobicity and elongation at break of the films. X-ray diffraction (XRD) analysis indicated good compatibility between gelatin and all three polysaccharides, while Fourier-transform infrared (FTIR) spectroscopy confirmed hydrogen bond interactions, leading to a more ordered film structure. These results show that polymer blending improves the functional properties of edible films, directly impacting their effectiveness in food preservation [34].

Given these findings, it was hypothesized that biopolymer-based films enriched with the antimicrobial agent nisin could improve the preservation of jumbo squid (*D. gigas*). Thus, the aim of this study was to evaluate the effects of biopolymer-based films made of alginate and gelatin enriched with Nisin/Na-EDTA on their mechanical, optical, and biodegradation properties, as well as their ability to reduce weight loss and maintain color stability during refrigerated storage of jumbo squid. Previous research has demonstrated that edible coatings can effectively extend the shelf life of seafood products, such as fish fillets and shrimp, by reducing microbial growth and moisture loss [26,35]. However, limited studies have explored their application in cephalopods, which are highly perishable due to their high protein and moisture contents. Therefore, this research seeks to bridge this gap by investigating the effectiveness of nisin-based films as a preservation strategy for high-value marine resources.

## 2. Materials and Methods

In this study, biopolymer-based formulations were initially cast into film form to serve as a model system for the controlled evaluation of their physicochemical and functional properties. Subsequently, the same formulation was applied as a surface coating on squid samples using the brushing method [28,36], demonstrating its potential as a practical alternative for food-contact applications.

### 2.1. Characterization of Biopolymer-Based Films

#### 2.1.1. Preparation of Biopolymer-Based Films

The biopolymer-based films were prepared using the casting method, following the procedure described by Abarca et al. (2022) with minor modifications [37]. The formulations for alginate-based, gelatin-based, and composite films were prepared as follows:

*Alginate-based film*. Control films were prepared from a solution containing 10% (*w*/*v*) sodium alginate and 10% (*w*/*v*) glycerol (as a plasticizer) in distilled water. The solution was stirred on a heating plate at 70 °C and 500 rpm for 2 h to ensure homogeneity and complete dissolution in the solvent.

*Gelatin-based film*. Control films were prepared separately using 10% (*w*/*v*) gelatins 220 or 280 Bloom, combined with 10% (*w*/*v*) glycerol in distilled water as reported by Abarca et al. (2022) [37]. The solutions were stirred at 60 °C and 1000 rpm for 2 h until homogeneity was achieved. It is important to highlight the differences between gelatin Bloom values, as they directly impact gel strength, setting behavior, and application. A 220 Bloom gelatin forms moderately firm gels, requires a slightly higher dosage, and sets at a moderate rate, making it suitable for soft-textured products such as dairy desserts and marshmallows. In contrast, a 280 Bloom gelatin exhibits higher gel strength, sets more rapidly, and achieves the same texture with less quantity, making it ideal for firmer applications such as gummy candies and hard capsules [38,39].

*Composite gelatin 220–alginate film (G:A ratio)*. Following an analysis of film and coating properties, gelatin 220 was selected for blending with alginate due to its superior water retention capacity (WRC), higher elongation at break (Y), and lower opacity (Op). These properties contributed to the development of an optimized composite film formulation. The optimized composite control film was prepared using 10% (*w*/*v*) sodium alginate, 10% (*w*/*v*) gelatin 220, 10% (*w*/*v*) glycerol, and 70% (*w*/*v*) distilled water. Meanwhile, the optimized composite active film was formulated with 10% (*w*/*v*) sodium alginate, 10% (*w*/*v*) gelatin 220, 10% (*w*/*v*) glycerol, 50% (*w*/*v*) distilled water, and 20% (*w*/*v*) Nisin/Na-EDTA. This latter formulation was chosen considering a previously tested quantity of Nisin/Na-EDTA of 20% (*w*/*v*) reported by Abarca et al. (2022) [37].

The active state of both polymeric-based films was achieved by incorporating the active agent Nisin/Na-EDTA, following the methodology outlined by Abarca et al. (2022) [37]. Briefly, Nisin/Na-EDTA solution at a concentration of 2500 (IU/mL) was prepared in accordance with the value obtained from the MIC. Then, it was incorporated into each formulation. The formulations were shaken on a hot plate (Scilogex model MS-H280-Pro, Scilogex, CT, USA) at 60 °C for 2 h with constant stirring at 1000 rpm.

Following homogenization, 70 g of the prepared solution was poured into glass Petri dishes and dried at 45 °C for 48 h.

#### 2.1.2. Mechanical Properties

The mechanical properties of the films were evaluated using a uniaxial tensile test at room temperature, according to the standard D882-12 [40]. Key mechanical parameters within the elastic limit, including the effective Young’s modulus (E_eff_), elongation at break (Y), and tensile strength (TS), were determined using a texture analyzer (EZ-LX, Shimadzu, Kyoto, Japan). The samples were securely clamped between grips, and the operational conditions were controlled using the Shimadzu TRAPEZIUM X v1.5.3 software. Stress/Strain curves were generated during extension at a rate of 5 mm/min, with an initial grip separation of 80 mm, a pre-load of 0.05 N, and a data acquisition frequency of 10 ms. The effective Young’s modulus was calculated using Equation (1).

(1) $$E_{eff}=\frac{stress}{strain}=\frac{\sigma }{\varepsilon }$$

where stress (σ) = F/A (Pa), and strain (ε) = ΔL/L_0_ (-) [41].

#### 2.1.3. Color Analysis

The chromatic parameters a*, b*, and L* were measured following the methodology described by Abarca et al. (2017) [42], with modifications. Briefly, the color values a* (-greenness/+redness), b* (-blueness/+yellowness), and L* (-black/+white) of the films were determined using a portable digital colorimeter (CR-400 series V.1.13, Konica Minolta, Tokyo, Japan) on random spots oriented perpendicularly to the film surface. A reference white calibration plate was employed for standardization (L* = 94.65, a* = −0.84, b* = 4.5). The CieLab total color difference (ΔE*) and whiteness index (WI) of the films were calculated for each sample (n = 15) using the respective equations:

(2) $$∆E*=\sqrt{{\left(∆L*\right)}^{2}+{\left(∆a*\right)}^{2}+{\left(∆b*\right)}^{2}}$$

(3) $$WI=100-\sqrt{{\left(100-L*\right)}^{2}+{\left(a*\right)}^{2}+{\left(b*\right)}^{2}}$$

where ΔL*, Δa*, and Δb* are the differences between the color values of the film modified and the control samples for lightness, red/green, and blue/yellow variations, respectively [42].

#### 2.1.4. Thickness, Opacity, and Transparency

The thickness of the films was measured using a digital micrometer (Digimatic ID-C112, Mitutoyo, Kawasaki, Japan) at fifteen random points on each film. Measurements were performed in triplicate, and the results were expressed as mean ± standard deviation according to Romina L. Abarca et al. (2023) [43]. Absorbance measurements were conducted using a spectrophotometer (UV-1650 PC, Shimadzu, Kyoto, Japan) at a wavelength of 600 nm, following the methodology of Abarca et al. (2022) and Irissin-Mangata et al. (2001) [37,44]. Film opacity (Op) and transparency (% T) were calculated as functions of film thickness and absorbance, according to Zhao et al. (2022) [45] using the following equations:

(4) $$Op=\frac{{Abs}_{600}}{\varepsilon }$$

(5) $$T\left(\%\right)=10^{\left(2-{Abs}_{600}\right)}$$

where Op = opacity (mm^−1^), Abs_600_ = absorbance at 600 nm, T = transparency (%), and ε = thickness of film (mm). Five repetitions were performed for each film [45].

#### 2.1.5. Biodegradability

Film degradation was evaluated following the methodology described by Oliveira Filho et al. (2019) [46], with modifications in the duration of the testing period. Organic soil contained in a plastic box was used as the degradation medium. Films were cut into 2 cm × 3 cm rectangles and placed on a mesh in direct contact with the soil. The humidity and temperature of the soil and surrounding environment were controlled and monitored throughout the experiment to ensure a stable relative humidity of 50%. Soil temperature was maintained at 21.9 ± 1.0 °C, while ambient temperature was 21.7 ± 1.5 °C. All experiments were conducted in triplicate. Film degradation was assessed by determining the mass of the films at each time point (M_t_) and expressing the results as a percentage of the initial mass (M_0_) [46].

### 2.2. Biopolymer-Based Coating: Evaluation of Weight Loss and Color

A specimen of squid (*Dosidicus gigas*) was used as a fresh food model to evaluate the effectiveness of surface application of biopolymer-based formulations. Squid samples were sourced from the local fisheries company TRIMAR (San Antonio, Chile). Only the mantle portion of the squid (fillet), both with and without skin, was utilized, and samples were prepared in standardized dimensions of 2 cm × 2 cm. The biopolymer-based formulations were applied to the surface of the squid using the brushing method because this offers a more uniform coating [28,36]. As a result, a continuous biopolymer-based coating was formed on the surface of the squid tissue. Color and weight loss were systematically monitored during storage at 4 °C over a seven-day period, following the methodology described by Dehghani et al. (2018) [35].

### 2.3. Statistical Analysis

A multifactorial analysis of variance (ANOVA) was conducted to evaluate the relationships between the factors and their levels. In this sense, the factors were the nature of films, including alginate, gelatin 220, gelatin 280, and gelatin 220–alginate ratio. The factors were studied at two levels: with and without the active agent Nisin/Na-EDTA. The variable responses were the Mechanical (Y, TS, E_eff_), Chromatic (L*, a*, b*, ΔE*), Opacity, Film degradation, and Weight loss (%) parameters of the product. Differences were declared to be statistically significant at *p* < 0.05, and Tukey’s test was applied. All analyses were conducted using the RStudio software v2024.09.0.

## 3. Results and Discussion

### 3.1. Mechanical Properties of Films

**Figure 1:**
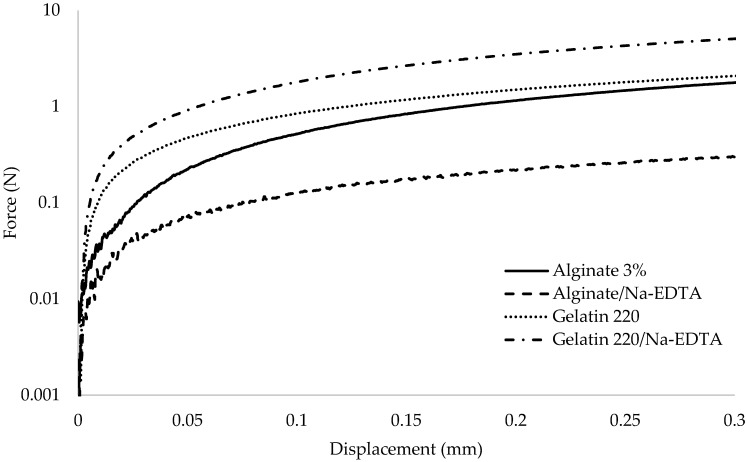
Mechanical behavior between force (N) and displacement (mm) for the tested biopolymer-based films.

The mechanical behaviors of films (Figure 1) and their characterizations are presented in Table 1, comparing those incorporating the active agent Nisin/Na-EDTA (active film) with their respective controls (without the active agent). The elongation at break (Y) ranged from 4.95 ± 0.50% to 65.13 ± 8.65%, with the lowest value observed for the alginate–control (4.95%) and the highest for the gelatin 280–active film (65.13%). The Y values demonstrated a significant dependence on the incorporation of the active agent into the matrix, with statistically significant changes observed (*p* ≤ 0.05), except for the G:A ratio.

For gelatin 220, the incorporation of the active agent Nisin/Na-EDTA resulted in a substantial decrease in Y values (*p* ≤ 0.05). In contrast, the incorporation resulted in a substantial increase in Y values for gelatin 280, alginate, and the G:A ratio compared with their respective controls (*p* ≤ 0.05), with increases of 1.76, 3.23, and 1.68 times, respectively. This behavior is likely attributed to matrix strengthening due to enhanced interactions between protein chains, as suggested by Rawdkuen et al. (2012) [47].

Said and Sarbon (2022) [48] reported various physical properties of gelatin-based films from different sources (mammalian, marine, and poultry), including Y values comparable to those obtained in this study [48]. Similarly, Bishnoi et al. (2022) [49] reported an elongation at break of 17.3 ± 2.1% for alginate/glycerin films, measured in the longitudinal direction [49]. Pranoto et al. (2005) [50] found Y values of 4.05% for alginate-based film in the absence of garlic oil, with a subsequent decrease as garlic oil concentration increased. These findings indicate that the evaluated films exhibited variations in their mechanical properties depending on the incorporation of the active ingredient [50].

Tensile Strength (TS) values ranged from 16.37 × 10^5^ to 88.63 × 10^5^ Pa for the control samples and from 2.80 × 10^5^ to 14.66 × 10^5^ Pa for the active films. The incorporation of the active agent Nisin/Na-EDTA into the matrix resulted in a significant reduction (*p* ≤ 0.05) in TS values across all film formulations; however, despite the incorporation of the active agent, the TS values of gelatin-based films exhibited a trend of increasing with Bloom.

The extent of TS reduction varied depending on the film composition, with the alginate-based film displaying the most pronounced decrease, an approximate tenfold reduction compared with its control. A similar decline in TS for alginate-based films upon the incorporation of an active agent was previously reported by Pranoto et al. (2005) [50]. TS reduction was also evident among the gelatin-based films, with gelatin 220 exhibiting a more substantial decrease (~6 times relative to the control) compared with gelatin 280 [51].

A distinct behavior was observed in the G:A ratio upon the incorporation of the active agent Nisin/Na-EDTA. This formulation had the most pronounced TS magnitude of the films tested, in contrast to the control, which had the lowest TS values. Farahnaky A. et al. (2014) [52] investigated the physical and mechanical properties of gelatin–clay nanocomposites, reporting that TS increased proportionally with clay content. Their findings also indicated a TS value of 2.19 ± 0.21 MPa for clay-free gelatin, which aligns with the magnitudes obtained in this study [52].

Furthermore, the incorporation of the active agent Nisin/Na-EDTA significantly influenced (*p* ≤ 0.05) the effective Young’s modulus (E_eff_). E_eff_ values ranged from 34.48 to 284.91 N/mm^2^ for control films and 31.64 to 69.50 N/mm^2^ for active films. As summarized in Table 1, E_eff_ decreased in almost all film formulations after the addition of the active agent, except for gelatin 220, which showed a significant increase (*p* < 0.05).

The mechanical responses of gelatin 220 and gelatin 280 varied with the addition of the active agent. In their pure state, gelatin 220 exhibited lower E_eff_ values than gelatin 280; however, upon incorporation of the active agent, this trend was reversed, with gelatin 220 displaying a higher E_eff_ than gelatin 280. Despite this shift, the E_eff_ value for the gelatin 220 control film in this study was consistent with the free-clay gelatin values reported by Farahnaky et al. (2014) [52].

The presence of the active agent Nisin/Na-EDTA resulted in a significant reduction in E_eff_ values for alginate-based films (*p* ≤ 0.05), with a nearly 9-fold decrease. This reduction is likely attributed to modifications in two glucuronic acid residues (GG), two mannuronic acid residues (MM), or the mannuronic–glucuronic acid residue (MG) block length, which play a crucial role in the formation of junction zones during gelation [35]. Additionally, this effect could be associated with conformational changes in the mannuronic/glucuronic (M/G) ratio in alginate or alterations in ionic strength resulting from the presence of EDTA [53].

The E_eff_ value for the alginate–control film was comparable to that reported by Kaklamani et al. (2014) [54] for a 3% (*w*/*w*) alginate concentration. However, their reported values were obtained in the presence of 1M Ca^+2^, highlighting potential differences in cross-linking conditions [54].

Therefore, the presence of the active agent Nisin/Na-EDTA induced significant changes in the mechanical properties of the films compared with their respective controls (*p* ≤ 0.05), irrespective of the biopolymer utilized. However, protein-based films exhibited superior mechanical properties compared with polysaccharide-based films. This behavior is likely due to the structural characteristics of proteins, which enable the formation of strong intermolecular covalent, ionic, and hydrogen bonds [51,55]. Moreover, crosslinking reactions are more common in proteins than in polysaccharides since proteins have more functional groups [54]. 

### 3.2. Thickness, Opacity, and Transparency of Films

Table 2 summarizes the thickness, opacity, and transparency values of the films tested. The results indicate that the thickness of gelatin-based films increased significantly (*p* ≤ 0.05) upon the incorporation of the active agent Nisin/Na-EDTA compared with their controls; specifically, gelatin-based films containing the active agent were approximately 1.42 and 1.39 times thicker than their counterparts without the active agent for gelatin 220 and gelatin 280, respectively. A similar trend was observed in the alginate-based film, which had a significant (*p* ≤ 0.05) increase in thickness of 1.7 times. In contrast, no significant variation (*p* > 0.05) was observed in the thickness of G:A ratio films. Moura-Alves et al. (2023) [56] reported sodium alginate films thicknesses ranging from 63.67 to 137.37 μm, with increasing values observed as olive leaf and laurel leaf extract were incorporated into the formulation, which support the results of this study.

In addition, substantial differences in thickness were detected between gelatin-based and polysaccharide-based films (*p* ≤ 0.05), regardless of the presence of the active agent Nisin/Na-EDTA. On average, gelatin-based films were approximately 6.4 times thicker than polysaccharide-based films. This increase in the thickness of gelatin-based films may be attributed to the higher solid content per surface unit incorporated by the active agent, as previously reported by Abarca et al. (2022) and Oliveira Filho et al. (2019) [37,46].

Opacity values of the polysaccharide-based films were significantly higher (*p* ≤ 0.05) than those of gelatin-based films, irrespective of the presence of the active agent Nisin/Na-EDTA. Opacity showed a broader range of values, between 0.391 and 3.138 mm^−1^ for control films and from 0.675 to 6.210 mm^−1^ for films containing the active agent. While gelatin-based films exhibited no statistically significant variation in opacity (*p* > 0.05) upon the incorporation of the active agent, polysaccharide-based films showed a notable increase in opacity. Therefore, gelatin-based films were significantly less opaque than polysaccharide-based films (*p* ≤ 0.05), regardless of incorporation of the active agent. The increased opacity observed in polysaccharide-based films could be attributed to the nonhomogeneous association or incomplete dispersion of components within the matrix [57].

Control films transmitted 60.84% to 68.04% of light at a wavelength of 600 nm. However, films containing the active agent Nisin/Na-EDTA exhibited a broader range of transparency values, between 24.09% and 67.48%. The transparency results (Table 2) further support the observation that gelatin-based films had a higher percentage of light transmission compared with polysaccharide-based films, which is in agreement with their lower opacity values. Among the tested formulations, alginate-based film exhibited the highest degree of light attenuation upon incorporation of the active agent Nisin/Na-EDTA, with a significant (*p* ≤ 0.05) reduction in transparency (2.5 times). This trend has previously been reported in the literature for films containing nanocomposite reinforcements [58,59].

**Figure 2:**
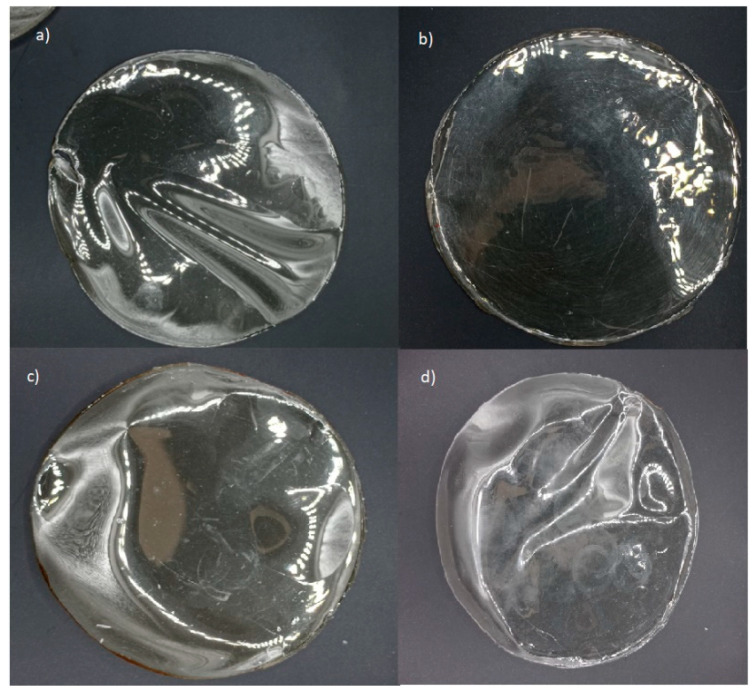
Edible gelatin-based films: (**a**) gelatin 220 without active agent; (**b**) gelatin 220 with active agent; (**c**) gelatin 280 without active agent; (**d**) gelatin 280 with active agent.

**Figure 3:**
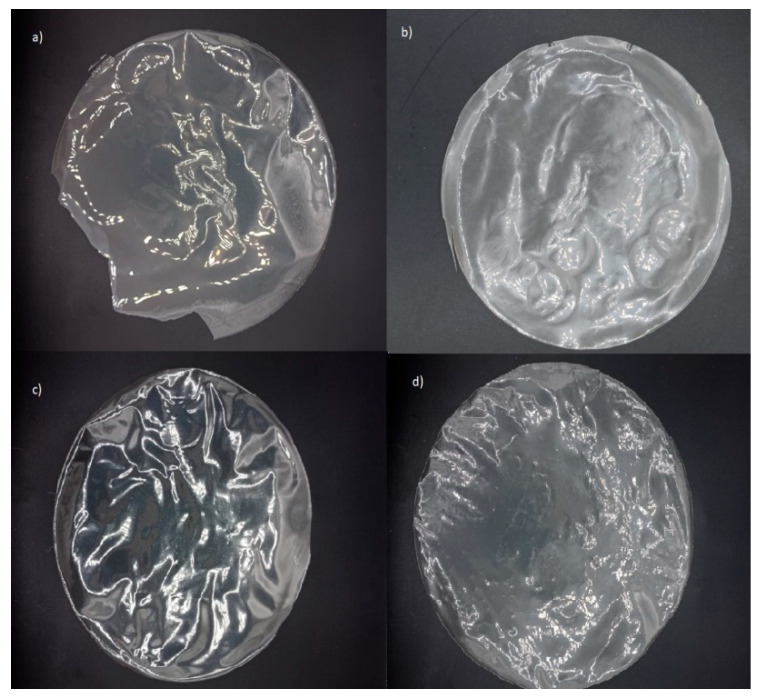
Edible alginate and G:A ratio-based films: (**a**) alginate without active agent; (**b**) alginate with active agent; (**c**) G:A ratio without active agent; (**d**) G:A ratio with active agent.

From a practical perspective, the high transparency of films enables consumers to visually inspect product details and assess the overall quality. In this regard, gelatin-based films effectively fulfill this function, irrespective of active agent incorporation (Figure 2 and Figure 3). However, their higher transparency may also facilitate accelerated photochemical reactions due to increased light transmission [58]. Conversely, alginate-based films, with their high opacity, could provide enhanced protective effects against light exposure when incorporating active agents.

### 3.3. Film Degradation

**Figure 4:**
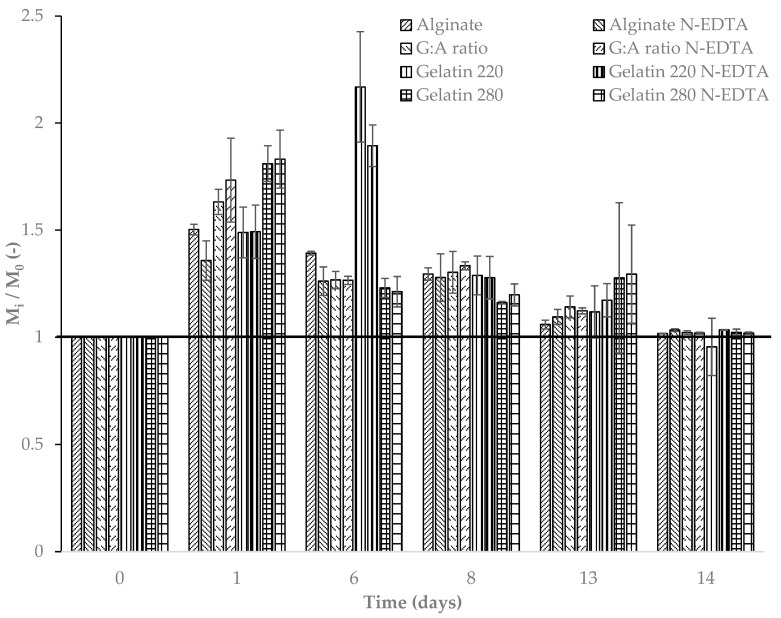
Non-dimensional variation in the weights of films over time at 50% relative humidity; Tsoil = 21.9 ± 1.0 °C and Tmedium = 21.6 ± 1.5 °C. The horizontal bold line is for reference.

Figure 4 illustrates the dynamic change in the weight of the film samples over time under ideal laboratory degradation conditions at 50% relative humidity. A significant (*p* ≤ 0.05) increase in film weight was observed across all samples on the first day of soil exposure, independent of film type.

**Figure 5:**
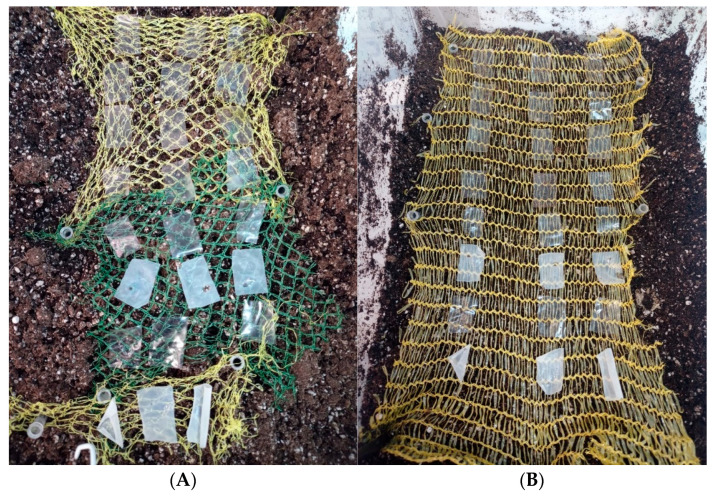
Degradation of edible biopolymer-based films over time at 50% relative humidity; Tsoil = 21.9 ± 1.0 °C and Tmedium = 21.6 ± 1.5 °C. (**A**) day 0; (**B**) day 8.

The initial increase in film weight during early exposure may be attributed to an adaptation period characterized by water absorption and swelling, as described by Azeredo and Waldron (2016) [60]. During this phase, weight gain ranged from 40% to 80% for all films tested. However, after six days of exposure, a significant reduction in weight was observed for all films except gelatin 220 in both its pure form and with the active agent Nisin/Na-EDTA (Figure 5). This delayed response in gelatin 220 is likely due to prolonged water absorption, which temporarily offsets the degradation process. By the eighth day, all films exhibited a noticeable decrease in weight compared with previous days (*p* ≤ 0.05), suggesting that the soil microbiota had begun to exert a more pronounced effect on film degradation under constant environmental conditions [37].

Among the tested films, polysaccharide-based formulations exhibited greater weight loss than protein-based films. Figure 4 illustrates the sustained reduction in the mass of the alginate-based film following its initial water absorption phase. Despite this common trend among the tested films, alginate and alginate–active films demonstrated lower water absorption and swelling compared with protein-based films. This behavior may be attributed to the pH of the surrounding medium, which may influence the retractive forces between the polymer chains [60].

The degradation reached statistically similar magnitudes across all film types by the fourteenth day (*p* > 0.05), indicating that the incorporation of the active agent Nisin/Na-EDTA did not significantly influence the final degradation rate. For longer exposure times, the trend suggests a continued degradation over time.

### 3.4. Assessment of Color and Weight Loss in Coated Fresh Squid

Raw squid samples, with and without skin, were coated with polymer-based formulations (with or without an active ingredient) and compared with uncoated samples in terms of weight loss (WL) after storage at 4 °C for 7 days (Table 3).

For squid samples with skin, WL values ranged from 46.15% to 63.63% for non-active coating and from 24.05% to 80.41% for coating containing the active agent Nisin/Na-EDTA. A significant difference (*p* ≤ 0.05) in WL was observed only when alginate and G:A ratio-based formulations were used as edible coating, likely due to the presence of the active agent Nisin/Na-EDTA within the structure; specifically, WL increased in the alginate-coated samples, whereas the opposite trend was observed for the G:A ratio coating containing the active agent Nisin/Na-EDTA.

When comparing coated samples with uncoated controls, most of them exhibited similar WL values (*p* > 0.05), except for the G:A ratio (with or without the active agent Nisin/Na-EDTA) and the alginate coating containing the active agent (*p* ≤ 0.05). Notably, the G:A ratio formulation exhibited the lowest WL values among all tested coatings, regardless of the presence of the active agent Nisin/Na-EDTA.

In contrast, squid samples without skin coated with biopolymer-based formulations exhibited a different trend in terms of WL values. The application of a coating—regardless of the presence of an active agent—did not significantly affect WL compared with the uncoated control (*p* > 0.05). The WL values for coated skinless samples ranged from 55.18% to 65.88%, representing a slight increase over the values observed for uncoated samples (62.88%).

These findings suggest that skinless squid samples were more susceptible to weight loss than their counterparts with skin, likely due to the absence of the natural protective barrier. Additionally, the limited water vapor barrier properties of the biopolymer-based formulations used in this study could have contributed to the increase in WL in the coated samples.

The comparison of WL values between squid samples with and without skin for each tested formulation generally did not reveal a clear trend, except for the G:A ratio-based coating, which exhibited a dependence on the natural squid skin for its protective efficacy.

Statistical analysis of WL as a function of the presence of skin showed significant differences (*p* ≤ 0.05) when the G:A ratio was used, regardless of the presence of the active agent Nisin/Na-EDTA. A similar trend was also observed for alginate formulation containing the active agent. In this context, G:A ratio-coated samples exhibited higher WL values in squid without skin, suggesting a potential synergistic protective effect between the natural skin and the G:A ratio polymer, independent of the active agent.

For the other biopolymer-based formulations, not statistically significant (*p* > 0.05) protective effect was observed in terms of WL reduction.

### 3.5. Color

**Figure 6:**
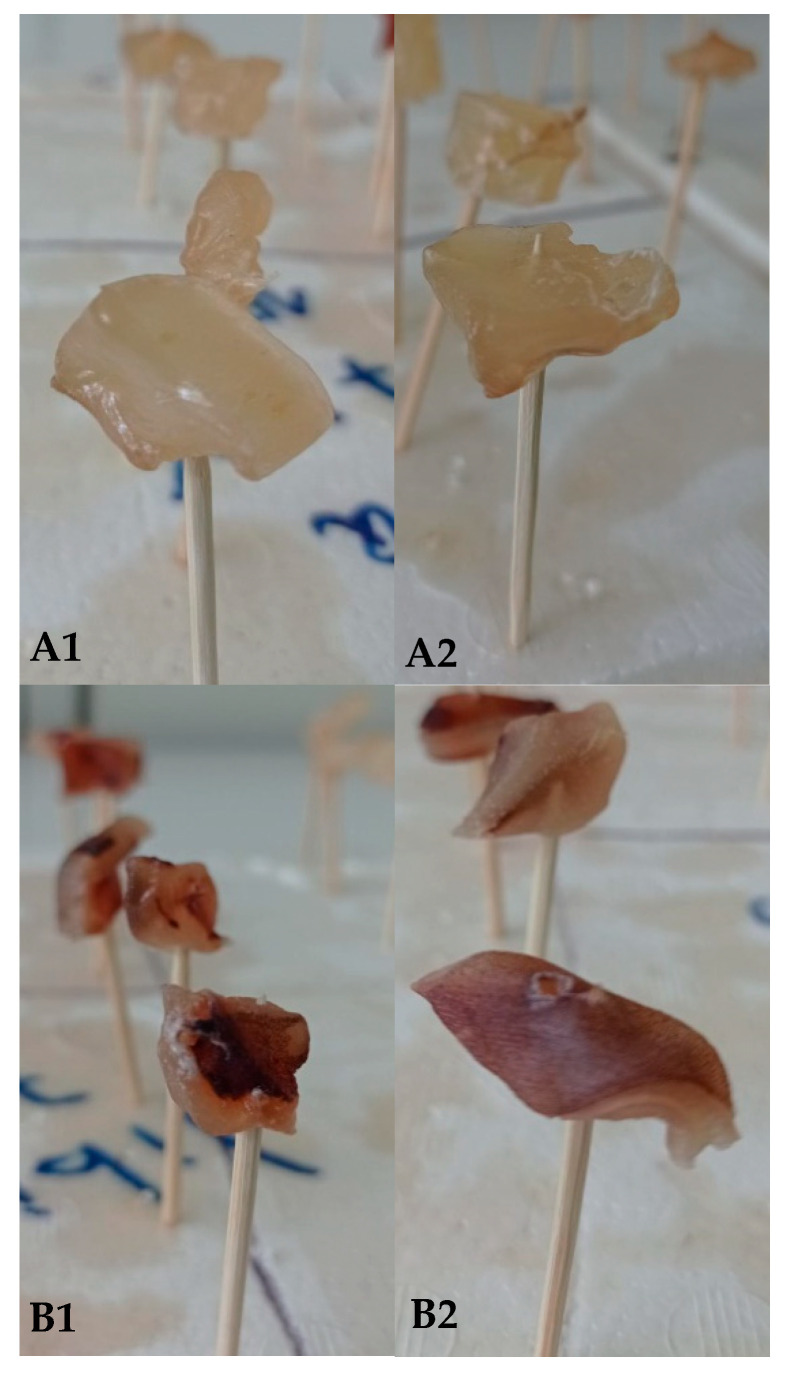
Effects of edible biopolymer-based film deposition on squid surfaces with (**B**) and without (**A**) skin at different times: (**A1**,**B1**) = day 0; (**A2**,**B2**) = day 8.

Table 4 presents the lightness (L*), chromaticity coordinates (a*: −120 green/+120 red and b*: −120 blue/+120 yellow), color difference (∆E*), and Whiteness Index (WI) values for the biopolymer-based formulations. In general, the incorporation of the active agent Nisin/Na-EDTA led to variations in color parameters to different extents (Figure 6).

Notably, the L* parameter remained largely unaffected (*p* > 0.05) by the presence of the active agent Nisin/Na-EDTA within the coating, with values ranging from 31.3 to 44.2% for squid with skin and from 51.2 to 57.1% for squid samples without skin (Table 4). This suggests that lightness was independent of the active agent’s presence. However, significant differences in L* values (*p* ≤ 0.05) were observed depending on whether the biopolymer-based formulation was applied to squid with or without skin, even for control squid. A similar trend for L* was reported by Luciano et al. (2021) [61] in gelatin-based films containing nisin at concentrations ranging from 0 to 112 mg nisin/g gelatin [61]. Likewise, Kaewprachu et al. (2018) [62] found no significant differences in L* values (*p* > 0.05) between gelatin films with and without nisin, both exhibiting L* values above 89.18% [62].

Similarly, a* and b* parameters exhibited no significant differences (*p* > 0.05) between formulations due to the presence of the active agent Nisin/Na-EDTA within the coating. However, gelatin-based coating containing the active agent Nisin/Na-EDTA showed statistically significant differences (*p* ≤ 0.05) in a* values compared with alginate-based coating for squid with skin. Despite this, the a* parameter did not show significant differences (*p* > 0.05) between coatings, regardless of the presence of the active agent. As observed for the L* parameter, statistical variations in a* were noted depending on whether the coating was applied to squid with or without skin (*p* ≤ 0.05).

For the b* parameter, the incorporation of the active agent Nisin/Na-EDTA into the coating, as well as the type of formulation applied to the squid, did not have a significant effect, in most cases (*p* > 0.05). However, in alginate-based coating, both with and without the active agent, b* values were significantly influenced (*p* ≤ 0.05) by the presence of squid skin.

The ΔE* values for squid samples with coating exceeded the established threshold for color differences perceptible by the human eye under all tested conditions [63]. For squid samples with skin, the ΔE* values were generally statistically similar to those of the control (*p* > 0.05). However, for squid samples without skin, significant differences in ΔE* values were observed, indicating a greater influence of the coating on color perception in these samples.

Specifically, gelatin-based coating applied to skinless squid exhibited ΔE* values that were statistically indistinguishable from those of the control, even with the incorporation of the active agent Nisin/Na-EDTA into the coating. In contrast, alginate-based coating, regardless of the presence of the active agent, had a detrimental effect on ΔE*, significantly altering the natural color perception of the squid surface as perceived by the human eye. Moreover, for skinless squid samples, the presence of the active agent Nisin/Na-EDTA had a positive effect by reducing ΔE* values compared with non-active coating, irrespective of the type of formulation utilized. These findings contrast with those reported by Luciano et al. (2021) [61], who observed an increase in ΔE* values in gelatin-based films with increasing nisin concentrations, reaching a maximum change of 5.3% at 112 mg nisin/g gelatin [61].

The Whiteness Index (WI) remained largely unchanged for squid samples with skin, regardless of the type of formulation applied or the incorporation of the active agent Nisin/Na-EDTA. WI values ranged from 30.2 to 41.9, with no statistically significant differences between treatments (*p* > 0.05). However, WI values for all coated samples were lower (indicating less whiteness) than the uncoated control squid, which may be attributed to a darkening of the squid surface. This latter observation is supported by variations in a* and b* chromatic parameters. The WI of squid with skin and coated exhibited a reduction of approximately 67% compared with the control sample. Similarly, WI values for skinless squid also showed a significant reduction, with statistically significant differences (*p* < 0.05) observed based on the presence of skin, irrespective of the type of coating applied. This was aligned with the WI value for control squid samples without skin, which naturally had higher L* values and represented a reduction of 25%. These findings contrast with those reported by Imran et al. (2012) [64], who reported an increase in WI values when nisin in nano-emulsion form was embedded into HPMC-based bioactive packaging films [64].

## 4. Conclusions

The addition of Nisin/Na-EDTA significantly modified the mechanical properties of all film types. Protein-based films exhibited superior mechanical strength relative to polysaccharide-based films, a phenomenon attributable to enhanced intermolecular interactions. In terms of optical characteristics, polysaccharide-based films showed higher opacity and lower transparency than gelatin-based films, particularly upon the incorporation of the active agent. Biodegradation studies revealed that while polysaccharide-based films degraded more rapidly, all formulations reached comparable degradation levels by day 14, indicating that the active agent did not influence long-term degradation behavior. Squid preservation trials highlighted the effectiveness of gelatin–alginate ratio coatings in minimizing weight loss, with or without the active agent. Although all coatings produced noticeable color changes, gelatin-based coating had a minimal impact, and the active agent reduced color variation in skinless squid. The Whiteness Index was unaffected in skin-on samples but decreased across coated samples due to surface darkening, especially in uncoated controls. This study showed that the presence of active agent acts in a complementary manner with the biopolymers-based formulations, particularly in terms of preserving the visual characteristics of squid.

These results highlight the potential of protein-based matrices for bioactive packaging in seafood products, offering a combination of enhanced mechanical and optical properties along with effective biodegradability and functionality for food packaging applications. However, certain gaps remain in this study; particularly concerning cytotoxicity and biocompatibility, which should be explored further in future investigations. Addressing these aspects is crucial to ensuring the safety and absence of any adverse effects on human health.

## Figures and Tables

**Table 1:** The mechanical properties of films composed of gelatin (280/220 Bloom), alginate, and G:A ratio presented as a function of the presence of the active agent Nisin/Na-EDTA. Control samples are films without the active agent.

Nisin/Na-EDTA Condition	Film	Y	TS	E_eff_
		(%)	(Pa) ×10^5^	(N/mm^2^)
non-active film	alginate	4.95 ± 0.50 ^A,x^	88.63 ± 9.46 ^A,x^	284.91 ± 0.67 ^A,x^
	gelatin 220	41.75 ± 5.05 ^A,y^	16.37 ± 1.18 ^A,y^	34.48 ± 8.37 ^A,y^
	gelatin 280	36.87 ± 7.52 ^A,y^	21.74 ± 17.94 ^A,y^	145.84 ± 8.32 ^A,z^
	G:A = 1:1	11.87 ± 1.87 ^A,z^	40.69 ± 0.87 ^A,z^	188.33 ± 9.69 ^A,z^
active film	alginate	16.03 ± 2.80 ^B,x^	7.98 ± 3.45 ^B,x^	31.64 ± 7.77 ^B,x^
	gelatin 220	26.39 ± 0.90 ^B,x^	2.80 ± 1.17 ^B,x^	69.50 ± 16.34 ^B,x^
	gelatin 280	65.13 ± 8.65 ^B,y^	7.46 ± 1.34 ^B,x^	48.15 ± 27.75 ^B,x^
	G:A = 1:1	19.98 ± 1.44 ^A,x^	14.66 ± 3.66 ^B,y^	41.87 ± 1.50 ^B,x^

Values expressed as mean ± standard deviation. Different capital letters (A, B) in the same column indicate significant differences (*p* ≤ 0.05) between non-active and active conditions with the same film. Different lowercase letters (x, y, z) in the same column indicate significant differences (*p* ≤ 0.05) among films for the same condition.

**Table 2:** Thickness, opacity, and transparency of films composed of gelatin (280/220 Bloom), alginate, and G:A ratio as a function of the incorporation of Nisin/Na-EDTA. Control samples are films without the active agent.

Nisin/Na-EDTA Condition	Film	Thickness ε (mm)	Opacity Op (1/mm)	Transparency T (%)
non-active film	alginate	0.05 ± 0.02 ^A,x^	3.14 ± 0.58 ^A,x^	60.84 ± 3.02 ^A,x^
	gelatin 220	0.35 ± 0.14 ^A,y^	0.39 ± 0.05 ^A,y^	68.05 ± 0.68 ^A,y^
	gelatin 280	0.37 ± 0.15 ^A,y^	0.43 ± 0.11 ^A,y^	63.97 ± 1.95 ^A,x^
	G:A = 1:1	0.06 ± 0.01 ^A,x^	2.49 ± 0.30 ^A,z^	61.22 ± 1.33 ^A,x^
active film	alginate	0.09 ± 0.02 ^B,x^	6.21 ± 1.13 ^B,x^	24.09 ± 2.77 ^B,x^
	gelatin 220	0.50 ± 0.06 ^B,y^	0.67 ± 0.04 ^A,y^	67.49 ± 0.68 ^A,y^
	gelatin 280	0.53 ± 0.12 ^B,y^	1.09 ± 0.24 ^A,y^	57.52 ± 5.23 ^B,z^
	G:A = 1:1	0.08 ± 0.01 ^A,x^	4.83 ± 0.55 ^B,z^	48.22 ± 1.88 ^B,w^

Values expressed as mean ± standard deviation. Different capital letters (A, B) in the same column indicate significant differences (*p* ≤ 0.05) between non-active and active conditions with the same film. Different lowercase letters (w, x, y, z) in the same column indicate significant differences (*p* ≤ 0.05) among films for the same condition.

**Table 3:** Weight loss (WL) of raw squid, with and without skin, coated with biopolymer-based formulations composed of gelatin (280/220 Bloom), alginate, and G:A ratio as a function of Nisin/Na-EDTA incorporation. Control samples are uncoated raw squid without the active agent.

Nisin/Na-EDTA Condition	Formulation	Weight Loss (WL) (%)	
		With Skin	Without Skin
Non-active film	alginate	63.63 ± 3.79 ^A,x,C^	59.77 ± 10.51 ^A,x,C^
	gelatin 220	68.69 ± 1.89 ^A,x,C^	65.17 ± 3.69 ^A,x,C^
	gelatin 280	65.70 ± 0.24 ^A,x,C^	64.21 ± 2.81 ^A,x,C^
	G:A = 1:1	46.15 ± 13.40 ^A,y,C,^*	65.88 ± 11.13 ^A,x,D^
Active film	alginate	80.41 ± 10.94 ^B,x,C,^*	64.29 ± 7.36 ^A,x,D^
	gelatin 220	54.98 ± 8.79 ^A,y,C^	55.18 ± 11.37 ^A,x,C^
	gelatin 280	71.28 ± 2.45 ^A,x,C^	61.34 ± 0.51 ^A,x,C^
	G:A = 1:1	24.05 ± 9.83 ^B,z,C,^*	59.44 ± 12.41 ^A,x,D^
Control	-	66.36 ± 8.62 *	62.88 ± 5.97 *

Values are expressed as mean ± standard deviation. Different capital letters (A, B) in the same column indicate significant differences (*p* ≤ 0.05) between non-active and active conditions with the same formulation. Different capital letters (C, D) in the same row indicate significant differences (*p* ≤ 0.05) between samples with and without skin. Different lowercase letters (x, y, z) in the same column indicate significant differences (*p* ≤ 0.05) among formulations for the same condition. * Indicates significant differences (*p* ≤ 0.05) among samples with respect to control.

**Table 4:** Chromatic parameter values of raw squid, with and without skin, coated with biopolymer-based formulations composed of gelatin (280/220 Bloom), alginate, and G:A ratio, as a function of Nisin/Na-EDTA incorporation. Control samples are uncoated raw squid without the active agent.

Nisin/Na-EDTA Condition	Formulation	L* (-)		a* (-)		b* (-)		ΔE* (-)		WI (-)	
		With Skin	Without Skin	With Skin	Without Skin	With Skin	Without Skin	With Skin	Without Skin	With Skin	Without Skin
non-active film	alginate	33.0 ± 2.0 ^A,x,C^	51.2 ± 4.9 ^A,x,D^	7.2 ± 3.7 ^A,x,C^	5.1 ± 0.1 ^A,x,C,^*	7.4 ± 2.2 ^A,x,C^	20.9 ± 2.9 ^A,x,D,^*	26.5 ± 2.6 ^A,x,C^	27.1 ± 1.1 ^A,x,C,^*	32.2 ± 1.5 ^A,x,C,^*	46.5 ± 3.4 ^A,x,D,^*
	gelatin 220	44.2 ± 10.1 ^A,y,C^	54.7 ± 2.7 ^A,x,D^	6.4 ± 2.6 ^A,x,C^	3.2 ± 0.6 ^A,x,D,^*	13.2 ± 5.8 ^A,x,C,^*	19.1 ± 2.2 ^A,x,C,^*	17.1 ± 6.5 ^A,y,C,^*	23.0 ± 2.1 ^A,x,D,^*	41.9 ± 8.2 ^A,y,C,^*	50.6 ± 1.7 ^A,x,D,^*
	gelatin 280	35.3 ± 4.3 ^A,x,C^	51.21 ± 1.4 ^A,x,D^	7.9 ± 1.2 ^A,x,C^	3.2 ± 1.5 ^A,x,D,^*	9.5 ± 2.5 ^A,x,C^	18.8 ± 4.2 ^A,x,D^	24.1 ± 3.9 ^A,x,C^	24.3 ± 3.6 ^A,x,C,^*	34.1 ± 3.8 ^A,x,C,^*	47.4 ± 0.9 ^A,x,D,^*
	G:A = 1:1	36.8 ± 3.5 ^A,x,C^	55.9 ± 1.6 ^A,x,D^	6.9 ± 0.1 ^A,x,C^	0.4 ± 0.6 ^A,y,D^	8.7 ± 1.4 ^A,x,C^	11.0 ± 1.0 ^A,y,C^	22.4 ± 3.8 ^A,x,C^	14.7 ± 2.7 ^A,y,D^	35.8 ± 3.1 ^A,x,C,^*	54.6 ± 1.6 ^A,y,D,^*
active film	alginate	31.3 ± 2.4 ^A,x,C^	55.3 ± 1.9 ^A,x,D^	10.2 ± 2.3 ^A,x,C^	3.5 ± 0.3 ^A,x,D,^*	6.8 ± 1.4 ^A,x,C^	20.9 ± 1.0 ^A,x,D,^*	29.0 ± 1.9 ^A,x,C^	24.2 ± 3.1 ^A,x,C,^*	30.2 ± 1.9 ^A,x,C,^*	50.5 ± 1.9 ^A,x,D,^*
	gelatin 220	36.2 ± 3.8 ^A,x,C^	57.1 ± 3.3 ^A,x,D^	5.2 ± 0.2 ^A,y,C^	3.2 ± 2.0 ^A,x,C,^*	7.9 ± 2.5 ^A,x,C^	19.3 ± 2.8 ^A,x,D,^*	22.6 ± 5.2 ^B,y,C^	22.2 ± 2.5 ^A,x,C,^*	35.5 ± 3.5 ^B,y,C,^*	52.7 ± 4.0 ^A,x,D,^*
	gelatin 280	32.8 ± 2.2 ^B,x,C^	55.4 ± 0.8 ^A,x,D^	9.7 ± 2.8 ^A,x,C^	1.7 ± 1.3 ^A,x,D^	8.7 ± 1.7 ^A,x,C^	14.7 ± 2.8 ^A,x,C^	27.2 ± 1.6 ^A,x,C^	18.6 ± 1.7 ^B,y,D^	31.5 ± 1.5 ^A,x,C,^*	52.9 ± 0.4 ^B,x,D,^*
	G:A = 1:1	34.3 ± 2.6 ^A,x,C^	54.3 ± 0.2 ^A,x,D^	5.4 ± 0.9 ^A,y,C^	1.1 ± 0.4 ^A,x,D^	7.7 ± 0.3 ^A,x,C^	13.7 ± 0.8 ^A,x,C^	24.5 ± 3.7 ^A,x,C^	18.0 ± 1.3 ^A,y,D^	33.7 ± 2.7 ^A,x,C,^*	52.2 ± 0.3 ^A,x,D,^*
Control		34.6 ± 0.6 ^C^	57.6 ± 1.3 ^D^	8.2 ± 0.5 ^C^	0.5 ± 0.5 ^D,^*	6.5 ± 0.7 ^C,^*	13.7 ± 1.7 ^D,^*	25.4 ± 1.6 ^C,^*	16.1 ± 2.2 ^D,^*	55.4 ± 1.3 ^C,^*	64.9 ± 1.9 ^D,^*

Values are expressed as mean ± standard deviation. Different capital letters (A, B) in the same column indicate significant differences (*p* ≤ 0.05) between non-active and active conditions with the same formulation. Different capital letters (C, D) in the same row indicate significant differences (*p* ≤ 0.05) between samples with and without skin for each chromatic parameter. Different lowercase letters (x, y) in the same column indicate significant differences (*p* ≤ 0.05) among formulations for the same condition. * Indicates significant differences (*p* ≤ 0.05) among samples with respect to control.

## Data Availability

The original data presented in the study are openly available in https://github.com/WladimirSilvaVera/Foods.git (accessed on 9 April 2025).
